# Our Decreasing Birth-Rate

**Published:** 1896-09

**Authors:** 


### OUR DECREASING BIRTH-RATE.

Figures lately compiled and published by Dr. J. S. Billings, perhaps our best authority on vital statistics, show that the birth-rate in America is on the decline, and this fact has not failed to attract the attention of the press—lay, medical, and scientific.

In 1880 the birth-rate per 1,000 in the United States was 30.95. In 1890 it was 26.68; a decrease of 4.27 per 1,000 of population in ten years. The decrease in the different States appears to have been nearly uniform. In Maine it was 3 per 1,000; in New York 1.65; in Pennsylvania, 3.30; in Indiana, 4.70; in Kansas, 5.67; in California, 3.72; in Louisiana, 5.50; in Texas, 9.47; in Kentucky, 4.90; in Georgia, 6.50; in Virginia, 7.76; and in other States in about the same proportion. The decrease was greatest in Massachusetts—11.67.

One cause of the decrease is said to be the drifting of the population toward the cities. Another, and probably more important factor is the desire of the *fin-de-siècle* woman to enjoy to the full the artificial and acquired "functions" of society, instead of the natural and instinctive functions of womanhood. Child-bearing, with all the self-sacrifices which this truly physiological condition imposes, is indeed not compatible with cotillons, euchre parties, and receptions. Therefore it is said that "woman's vanity threatens the race."

The declining birth-rate in France is well known, and it has excited some uneasiness among that patriotic people. Recourse has been had to legislation, societies have been formed to consider and devise ways and means of holding up the birth-rate, premiums have been offered on large families, etc., but the babies are not forthcoming.

It is said that a diminishing birth-rate has presaged the decline and fall of nations, notably Greece and Rome. Is a similar destiny in reserve for France and America?

